# Direct Current-Induced Calcium Trafficking in Different Neuronal Preparations

**DOI:** 10.1155/2016/2823735

**Published:** 2016-12-15

**Authors:** Andrzej Wieraszko, Zaghloul Ahmed

**Affiliations:** ^1^Department of Biology, The College of Staten Island/City University of New York, 2800 Victory Boulevard, Staten Island, NY 10314, USA; ^2^Department of Physical Therapy, The College of Staten Island/City University of New York, 2800 Victory Boulevard, Staten Island, NY 10314, USA

## Abstract

The influence of direct current (DC) stimulation on radioactive calcium trafficking in sciatic nerve in vivo and in vitro, spinal cord, and synaptosomes was investigated. The exposure to DC enhanced calcium redistribution in all of these preparations. The effect was dependent on the strength of the stimulation and extended beyond the phase of exposure to DC. The DC-induced increase in calcium sequestration by synaptosomes was significantly reduced by cobalt and rupture of synaptosomes by osmotic shock. Although both anodal and cathodal currents were effective, the experiments with two electrodes of different areas revealed that cathodal stimulation exerted stronger effect. The exposure to DC induced not only relocation but also redistribution of calcium within segments of the sciatic nerve. Enzymatic removal of sialic acid by preincubation of synaptosomes with neuroaminidase, or carrying out the experiments in sodium-free environment, amplified DC-induced calcium accumulation.

## 1. Introduction

For the past twenty years, there has been a growing interest in noninvasive methods to stimulate the nervous system. One of them, rediscovered over fifteen years ago [1, 2], involves passing of the polarized, low-intensity current (1–3 mA) via electrodes located either on the scalp or in the proximity of the spinal cord. The effects occur relatively fast and often outlast the period of stimulation [3–6]. The consequences of DC stimulation are complex and seem to affect differently axonal [7] and synaptic components of the nervous system [4, 6, 8, 9]. The influence on the axon is presumably mediated by depolarization or hyperpolarization of the membrane [7, 8, 10]. Initial investigations of synaptic effects determined that hyperpolarization and depolarization increased and reduced the amount of neurotransmitter at neuromuscular junction, respectively [11, 12]. Recent research revealed that synaptic modulation exerted by DC is likely to occur via LTP- and LTD-like mechanisms implicated in synaptic plasticity [13–15]. While DC stimulation of the brain helps to ameliorate symptoms of psychological disorders [16–20], the exposure of the spinal cord to DC modulates spontaneous activity of the neurons [5, 8, 21] changing corticospinal interactions [5, 8, 21]. Those modulations are likely responsible for DC-induced improvement in the recovery after spinal cord injuries [5, 6, 9, 22–25]. It is well established that alteration of neuronal functions relies heavily on the spatially organized calcium signaling and changes in intracellular calcium concentration [26–28]. The release of neurotransmitters [29], neuronal migration [30], synaptic plasticity [31, 32], and organization of neuronal networks [33] are just a few specific examples of the processes which require very strict and precise control of calcium homeostasis and distribution within the neuron. Indeed, individual calcium channels are advantageously localized in the proximity of other signaling molecules (e.g., glutamate receptors, Ca^2+^ channels, and nitric oxide synthase), organized along the internodal axolemma under the myelin sheath in discrete “axonal nanocomplexes” [34]. Although overactivation of nanocomplexes during disease can lead to an excessive increase in intracellular Ca^2+^ [35, 36], the influence of DC on these nanocomplexes and subsequent discreet elevation of intracellular calcium concentration could contribute to plasticity of neuronal networks, as observed in the CNS during induction of LTP and LTD [37]. Thus, one can assume that the influence of DC stimulation on CNS is at least partially mediated by modulation of the intracellular calcium concentration. Indeed, as reported by Ranieri and collaborators [14], the intensity of LTP was significantly changed by the exposure to DC. In subsequent, parallel experiments, Ahmed and Wieraszko [6] reported DC-induced modulation of the release of glutamate, a major neurotransmitter involved in induction and maintenance of LTP [38]. Conceding a strong influence of DC on neuronal activity in the brain [15, 39, 40] and spinal cord [5, 8, 9, 21], current investigation was focused on the influence of DC exposure on calcium trafficking in neuronal preparations in vivo and in vitro.

The changes in intracellular calcium concentration can be detected with either fluorescent probes [41, 42] genetically encoded calcium indicators [43] or radioactive tracer [44]. Most of the fluorescent probes enter the cell as hydrophobic esters and become charged in the cytoplasm. Therefore, their intracellular location and movement can be significantly altered by subsequent exposure to DC. Genetically encoded calcium indicators represent very promising but challenging method still under development [41, 42]. As confirmed by Islam and collaborators [44], the changes in calcium distribution in neuronal tissue can be estimated with radioactive calcium. However, their [44] radiographic quantitative data analysis is less reliable than determination of labelled calcium in the tissue prepared for qualitative scintillation counting. Additionally, the usage of autoradiography would be difficult for some of the preparations used and compared in our experiments. Therefore, as a method of choice in determination of the influence of DC exposure on the translocation of calcium in different preparations in vivo and in vitro, we used radioactive calcium. Its relocation from the incubation medium into the cellular compartment can be reliably and reproducibly followed both in vivo and in vitro.

## 2. Methods

### 2.1. In Vivo Experiments

**Figure 1:**
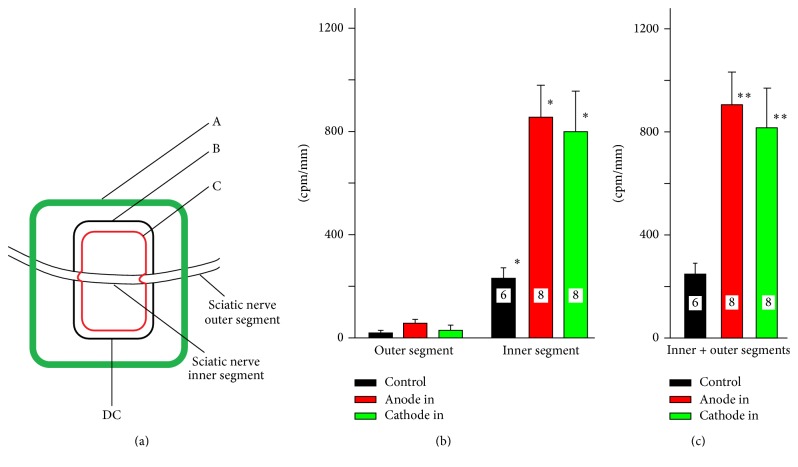
The accumulation of ^45^Ca^2+^ by DC-stimulated sciatic nerve in vivo; (a) an experimental set up. The exposed segment of the sciatic nerve was surrounded by the barrier made of hydrophobic petroleum jelly/silicone oil mixture which formed the external pool (A). The second smaller (internal) pool (B) was made of the same petroleum jelly on the stimulation plate (C) inserted underneath the sciatic nerve. The stimulation plate (15 × 20 mm) was insulated from the tissue by the rectangular piece of the rubber located under the stimulation plate (omitted for the clarity from the picture). This experimental arrangement allowed keeping outer and inner segments of the sciatic nerve in different chambers which were electrically isolated from each other except for the connection made by the nerve itself. (b) ^45^Ca^2+^ accumulated in the inner and outer segments (cpm/mm) in control experiments (no stimulation, black), and when either anode (red) or cathode (green) was connected to the stimulation plate (^*∗*^ *p* < 0.001, one-way ANOVA followed by Dunn's test). (c) The total accumulation of ^45^Ca^2+^ in inner and outer segments (^*∗∗*^ *p* < 0.001, Mann–Whitney* U* Sum Rank test, control versus “anode in” and “cathode in” for combined segments). In this legend and all subsequent figure legends, the numbers inside of the bars indicate the number of separate experiments.

We have used three different neuronal preparations to verify the hypothesis that exposure of neuronal tissue to DC stimulation modifies the concentration of intracellular calcium. As the first initial approach, we used the in vivo model system used by us previously [8]. Following exposure of the sciatic nerve in anesthetized mice, the petroleum jelly/silicone oil mixture was applied on the tissue to form a Ringer's solution-containing chamber with segment of the nerve inside of it (Figure 1(a), according to [8], modified). One rectangular electrode made of stainless steel (7 mm × 15 mm) was placed below the sciatic nerve which was insulated from the rest of the body by piece of rubber located underneath the electrode. The DC reference electrode was attached to the abdominal skin. The petroleum jelly/silicone oil mixture was used to create a second, smaller centrally located chamber on surface of stimulating plate (Figure 1(b)). The larger and smaller chambers were electrically insulated from each other with jelly/silicone oil mixture except for connection through the nerve. The segment of the sciatic nerve inside of smaller chamber and two segments of the sciatic nerve outside of the small chamber were termed as “inner” and “outer segments,” respectively. For technical reasons only the pieces of the sciatic nerve from inner chamber and outer chamber distal to stimulating electrode were taken for subsequent analysis. The plate electrode was connected to either anodal or cathodal DC stimulator (ActivaDoseII, Iontophoresis delivery unit). ^45^Ca^2+^ has been added to the central chamber to achieve the final concentration of 300 nM. Control experiments revealed that the seal was effective and there was practically no leak of radioactivity. Following stimulation period (0.8 mA, 20 min), the inner and outer segments of the sciatic nerve were dissected out, washed superficially in 100 ml of cold Ringer's solution, dried on the filter paper, weighted, measured in length, and homogenized in glass/glass homogenizer. The radioactivity of each homogenate was evaluated with the scintillation counter (Beckman Coulter LS 6500) and expressed in counts per minute per mm of the nerve (cpm/mm).

### 2.2. In Vitro Experiments

#### 2.2.1. The In Vitro Experiments on Dissected Segments of Sciatic Nerve

**Figure 2:**
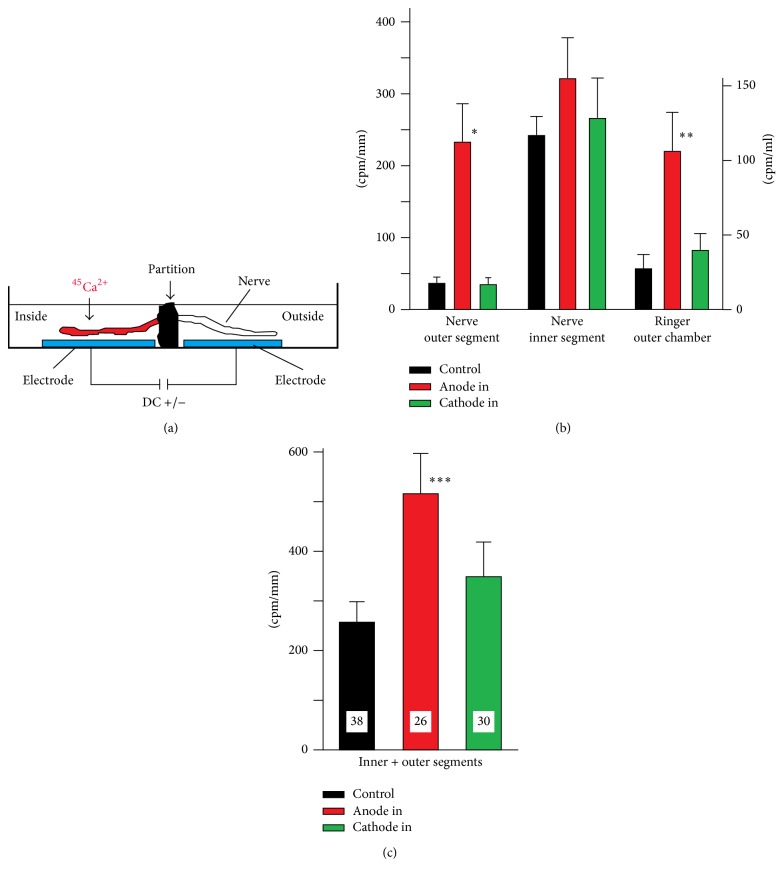
The accumulation of ^45^Ca^2+^ by the DC-stimulated sciatic nerve in vitro. (a) An experimental setup. The Petri dish (3 cm in diameter) was divided into two parts with partition made of hydrophobic petroleum jelly/silicone oil mixture. There was a stainless steel electrode placed at the bottom of each part (6 × 10 mm). The segment of the sciatic nerve was extended through the partition. The only electrical connection between two parts of the Petri dish was through the sciatic nerve. The electrodes were connected to DC power supply delivering 0.1 mA for 3′. The reference electrode was much closer (approximately 8–10-fold) than in in vivo experiments. Therefore, to compensate for the distance and equalize experimental conditions, the intensity of stimulation for these experiments was adjusted accordingly. ^45^Ca^2+^ was always added to the same part called “inner chamber.” (b) ^45^Ca^2+^ accumulation in the inner and outer segments exposed to either anodal or cathodal stimulation delivered to inner chamber. The anodal DC stimulation induced statistically significant increase in ^45^Ca^2+^ accumulation in the outer segment (^*∗*^ *p* < 0.001, Mann–Whitney* U* test) and in Ringer's solution collected from outer chamber (^*∗∗*^ *p* < 0.03, Mann–Whitney* U* test). The anodal and cathodal stimulations induced a similar increase in the segment of the nerve located in the inner chamber, although those increases were not statistically significant. Note the increase in the concentration of ^45^Ca^2+^ in the outer chamber (right side of Figure 1(b)). This diagram has two separate scales to express the amount of ^45^Ca^2+^ in the nerve (cpm/mm, left scale) and the concentration of ^45^Ca^2+^ in the Ringers of the outer chamber (cpm/ml, right scale). (c) The total accumulation of ^45^Ca^2+^ in the inner and outer chambers; ^*∗∗∗*^ *p* < 0.001 as compared with control, and *p* < 0.036 as compared with “anode in”; Mann–Whitney* U* test.

As previously demonstrated by us, sciatic nerves maintained in vitro according to our procedure preserve evoked activity [5]. The nerve dissected out from anesthetized animal (2-3 cm) was preincubated at least one hour in Ringer's solution prior to experimental procedure to allow for sealing of cut, axonal ends [45]. The influence of DC stimulation on ^45^Ca^2+^ accumulation and distribution within stimulated sciatic nerve was tested in a specially designed chamber (Figure 2), which was divided into two separate small pools with partition made of jelly/silicone oil mixture used for in vivo experiments as well. The only electrical connection between these two chambers was possible by the tissue of sciatic nerve which extended from one chamber to the other penetrating through insulating barrier (Figure 2(a)). Each pool had an electrode (10 × 6 mm) at the bottom and these electrodes were connected to either anodal or cathodal currents generated by ActivaDoseII, Iontophoresis delivery unit. ^45^Ca^2+^ (^45^CaCl_2_, PerkinElmer, approximately 300.0 nM in the chamber) was always added to the chamber termed “inside.” Following DC stimulation (3′, 0.1 mA), the nerve was cut at the partition, and the amount of ^45^Ca^2+^ was determined in the nerve as described above for in vivo experiments. The distance between two electrodes used for in vitro stimulation was much shorter than the same distance in in vivo experiments. Therefore, to make the results more comparable, the intensity of the current was adjusted accordingly. The amount of ^45^Ca^2+^ in Ringers of the outside chamber was also evaluated.

#### 2.2.2. Segments of the Spinal Cord

**Figure 3:**
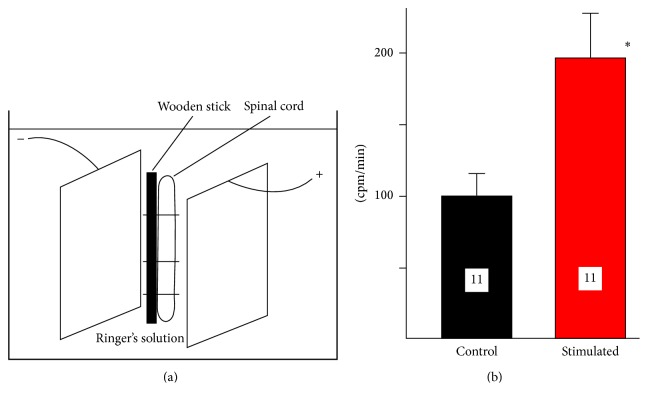
The ^45^Ca^2+^ accumulation by the segments of the spinal cord stimulated in vitro by two electrodes of the same size. (a) An experimental setup. Two stainless steel plates (30 × 7 mm) were placed 9 mm apart inside of the plastic tube and connected to the source of DC. The segment of the spinal cord, attached to the wooden stick, was placed between two electrodes. (b) Accumulation of ^45^Ca^2+^ in the segment of the spinal cord (in cpm/mm) following 3 mA, 3′ stimulation. The increase in stimulated spinal cord (190.9%) is statistically significant (^*∗*^ *p* < 0.029, *t*-test).

The segments of the spinal cord (2-3 cm long) have been dissected from anesthetized animals, attached to the wooden stick as in our previous experiments [6], and placed in the plastic tube containing identical, stainless steel electrodes (30 × 7 mm; 210 mm^2^ each, the distance between electrodes was 9 mm, Figure 3(a)).

#### 2.2.3. Synaptosomes

Synaptosomes were obtained from cerebral cortex according to modified procedure described by Sawynok and collaborators [46]. The tissue has been homogenized in 0.23 M sucrose (1 : 10, tissue/sucrose ratio) in the teflon/glass homogenizer and centrifuged for 10 min at 5000 ×g. The pellet has been discarded and the supernatant was centrifuged for 20′ at 19000 ×g. The supernatant was discarded and the pellet (synaptosomal fraction) was suspended in Ringer's solution and used for the experiments. This procedure would yield suspension of synaptosomal vesicles of 0.5–0.6 *μ*m in diameter. The synaptosomal fraction (1000 *μ*l corresponding to 250 mg of wet tissue) was transferred to the plastic tube used for the experiments with the segments of the spinal cord (see above). The synaptosomes were stimulated after addition of ^45^Ca^2+^ (final concentration was approximately 300 nM) for 3′ with DC varying in control experiments from 1 to 4 mA, while ^45^Ca^2+^ was already present in the solution. In all subsequent experiments, synaptosomes were stimulated for 3′ with 3 mA current. In some experiments, brain synaptosomes were stimulated in the presence of 5 mM cobalt. In alternative sets of experiments, ^45^Ca^2+^ was not present in the solution during the stimulation, but it was added 6 hrs after cessation of stimulation. The impact of modified composition of Ringer's solution by substituting NaCl with osmotically equivalent choline chloride was also tested. The influence of partial, enzymatic removal of sialic acid from the surface of brain synaptosomes on DC-induced ^45^Ca^2+^ accumulation was assessed in separate set of experiments. The mixture of synaptosomes was incubated for 3 hrs at 33°C with neuroaminidase from Vibrio Cholerae (0.2 U/ml; Sigma). Then, the accumulation of ^45^Ca^2+^ by control and DC-stimulated synaptosomes was evaluated. It has been of paramount importance to determine if the integrity of synaptosomes was compromised by DC stimulation. Therefore, the DC-induced ^45^Ca^2+^ accumulation was compared between stimulated, control fraction of synaptosomes and fraction of synaptosomes exposed to osmotic shock. It is well known that synaptosomes burst in hypoosmotic environment transforming fraction of synaptosomes into suspension of membranes [47]. The synaptosomal suspension was transferred from isoosmotic Ringer's solution to water which would cause hypoosmotic destructions of synaptosomes. At the completion of all DC stimulation experiments, the synaptosomal suspension was filtered under vacuum (Whatman GF/B filters, soaked for 1 hr in 0.1% polyethylenimine solution before experiment), and the radioactivity remaining on the filters was counted in the scintillation vials after addition of scintillation fluid (Beckman Coulter LS 6500).

#### 2.2.4. Stimulation with the Electrodes of Different Sizes

It is well documented that polarity of direct current is crucial for its effect [8, 48, 49]. Therefore, in separate experiments, the influence of anodal and cathodal stimulation on calcium accumulation by the spinal cord or synaptosomal suspension was tested in the tube with two electrodes of different sizes (Figure 6(a)). While one of the electrodes consisted of stainless steel plate (210.00 mm^2^), the second electrode was made of stainless steel wire (11.75 mm^2^). The distance between these two electrodes was 9 mm (Figure 6(a)). Following the addition of ^45^Ca^2+^ (300 nM in the chamber) and stimulation (3 mA, 3′), the fraction of synaptosomal suspension or segments of the spinal cord were superficially washed and homogenized and the amount of radioactivity was determined as described above. The type of test used for statistical analysis and calculated probability are illustrated in Table 1.

## 3. Results

### 3.1. Axonal, DC-Induced Accumulation of ^45^Ca^2+^ In Vivo

In vivo experiments revealed that exposure to DC considerably enhanced accumulation of ^45^Ca^2+^ by stimulated segment of the sciatic nerve located directly on the stimulation plate (inner segment, Figure 1). We used the term “anodal” or “cathodal stimulation” when the anode or cathode was connected to this plate, respectively. Anodal stimulation was more efficient in enhancing calcium accumulation. Over 90% of accumulated calcium could be recovered in the inner segment, although there was some accumulation in the outer segment as well, especially in the case of anodal stimulation. Since there was no leak of Ringer's solution between the inner and the outer chambers, the only source of ^45^Ca^2+^ in the outer segment could be redistribution of ^45^Ca^2+^ within the axon [28]. This relocation seemed to be more extensive in the case of anodal stimulation. The presence of ^45^Ca^2+^ in the outer segments indicates that calcium, accumulated by the nerve under DC influence, was electrophoretically driven along the length of the axon further away from the site of the exposure towards anodal or cathodal end of the nerve. There was some negligible relocation of ^45^Ca^2+^ from inner to outer chamber in control experiments (no stimulation) as well (Figure 1(b)). The difference between anodal and cathodal stimulation was statistically insignificant, although it became greater when the amounts of calcium accumulated by inner and outer segments were added together (Figure 1(c)). Anodal stimulation was significantly more efficient in stimulation of ^45^Ca^2+^ accumulation than cathodal stimulation. Statistical analysis (one-way ANOVA followed by Dunn's test, ^*∗*^ *p* < 0.001) revealed that the difference between inner control segment versus “anode in” and “cathode in” was statistically significant at *p* < 0.008 and *p* < 0.03, respectively (Mann–Whitney* U* Rank Sum test). The difference between control and “anode in” and “cathode in” for combined segments (C) was also statistically significant (^*∗∗*^ *p* < 0.001; Mann–Whitney* U* Sum Rank test). It should be emphasized that there were several factors which could influence recorded data. Some of those factors which were very difficult to control included fluctuation in the temperature of the exposed, dorsal part of the animal's body, the amount of the moisture in the vicinity of the plate, pulsation of the blood vessels, and movements due to breathing.

### 3.2. DC-Induced Accumulation of ^45^Ca^2+^ by Axons In Vitro

The results of the subsequent in vitro experiments essentially reinforced the data obtained in vivo. Those experiments were designed differently (Figure 2(a)) and allowed for much more precise control of experimental conditions. Clearly, anodal stimulation was more effective in enhancing of ^45^Ca^2+^ accumulation in the nerve segment (^*∗*^ *p* < 0.001, Mann–Whitney* U* test, Figures 2(a) and 2(b)) and in the chamber (*p* < 0.03, Mann–Whitney* U* test, Figures 2(a) and 2(b)). The anodal current not only facilitated significant relocation of calcium ions within the axon but markedly increased the displacement of calcium ions from the nerve to the outer chamber (Figure 2(b)).  Figure 2(c) depicts combined results obtained from inner and our chambers (*p* < 0.036, Mann–Whitney* U* test). The control, nonstimulated segments showed relatively high accumulation of ^45^Ca^2+^ which might be the result of incomplete sealing of cut, exposed ends of the nerve [45].

### 3.3. DC-Induced Accumulation of ^45^Ca^2+^ by Spinal Cord In Vitro

The tissue of isolated spinal cord is too delicate and fragile to be reliable and reproducibly placed in the chamber designed for sciatic nerve (Figure 2). Therefore, the spinal cord attached to the wooden stick for better stability (Figure 3(a)) was tested only for total accumulation of ^45^Ca^2+^ during exposure to DC (3′, 3 mA). Like the sciatic nerve, the isolated segments of the spinal cord, located between two electrodes of the same size, accumulated more ^45^Ca^2+^ when exposed to DC stimulation (190.9%, ^*∗*^ *p* < 0.029, *t*-test, Figure 3(b)). The effect was significant, although experimental design did not allow distinguishing between the influences of anodal and cathodal currents.

### 3.4. DC-Induced ^45^Ca^2+^ Accumulation by Synaptosomes

**Figure 4:**
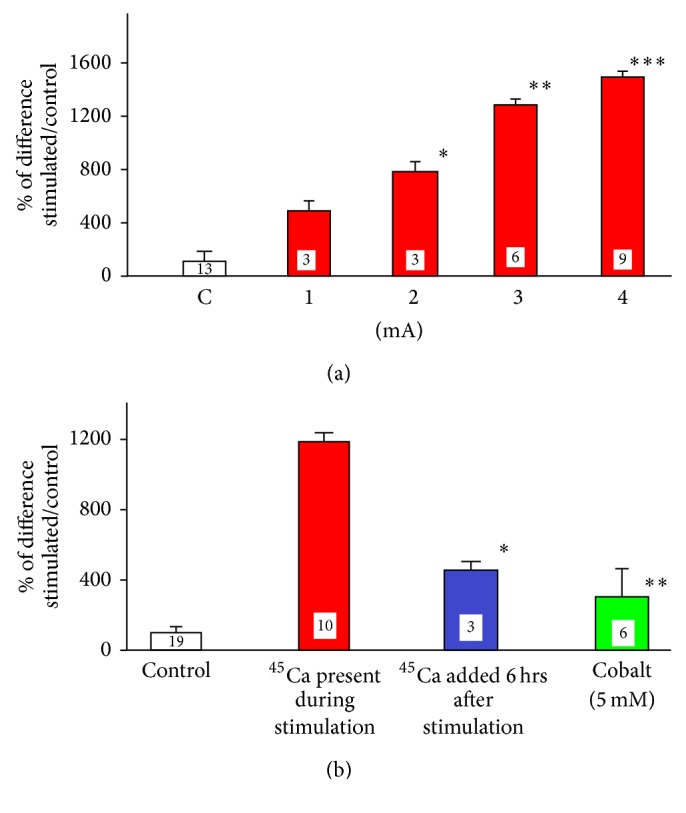
The influence of different experimental conditions on ^45^Ca^2+^ accumulation by synaptosomes. (a) Accumulation of ^45^Ca^2+^ by fractions of synaptosomes stimulated for 3 min by DC ranging from 1 to 4 mA. The results are presented as % of the difference between stimulated and nonstimulated preparations. ANOVA followed by Holm-Sidak, *p* < 0.001; ^*∗*^1 versus 2 mA n.s.; ^*∗∗*^2 versus 3 mA, *p* < 0.001; ^*∗∗∗*^3 versus 4 mA, *p* < 0.04, *t*-test. (b) The accumulation of by stimulated synaptosomal suspension while ^45^Ca^2+^ was either present in the tube during stimulation (red) or added to the tube 6 hrs after cessation of stimulation (blue); ^*∗*^ *p* < 0.003, *t*-test, as compared to nonstimulated control. The accumulation of ^45^Ca^2+^ in the presence of 5 mM cobalt (green); ^*∗∗*^ *p* < 0.001, *t*-test, compared to synaptosomes stimulated in the presence of ^45^Ca^2+^.

**Figure 5:**
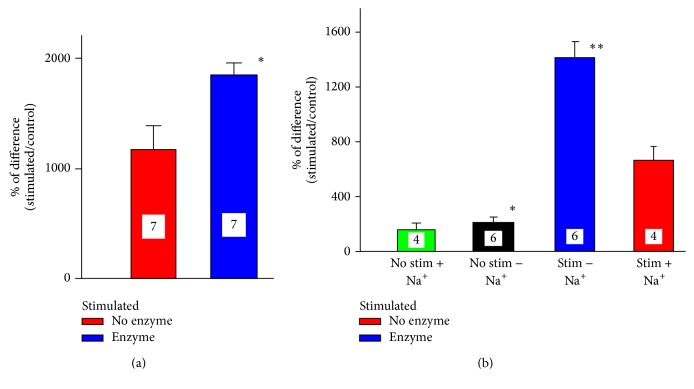
The modification of ^45^Ca^2+^ accumulation by synaptosomes either treated for 3 hrs with neuroaminidase (enzyme) or incubated in sodium-free Ringer's solution. (a) Treatment of synaptosomes with neuroaminidase (0.2 U/ml) significantly increased DC-evoked ^45^Ca^2+^ accumulation, as compared with nontreated ones and also by stimulated controls; ^*∗*^ *p* < 0.006, *t*-test. (b) The omission of sodium ions from Ringer's solution enhances ^45^Ca^2+^ accumulation in DC-stimulated and nonstimulated synaptosomes. The control, nonstimulated synaptosomes accumulated more ^45^Ca^2+^ in Na^+^-free Ringer's solution (black bar), than in the presence of sodium (green bar); ^*∗*^ *p* < 0.001, ANOVA followed by Dunn's test; ^*∗∗*^ *p* < 0.001. Stimulated synaptosomes accumulated less calcium in the presence of sodium (red bar), than in Na^+^-free solution (blue bar), *p* < 0.029, *t*-test.

In the pursuit of further characterization of the influence of DC stimulation on ^45^Ca^2+^ trafficking, we used fraction of synaptosomes which can be prepared and tested in a very reproducible manner. Similarly as sciatic nerve and the spinal cord, synaptosomes also accumulated more ^45^Ca^2+^ when exposed to DC. As depicted in Figure 4(a), increasing the intensity of the stimulating current induced proportional increase in ^45^Ca^2+^ accumulation. Although 4 mA was the most effective within the tested range of intensities, 3 mA were employed in most of the other experiments. Synaptosomes damaged by osmotic shock and stimulated subsequently with DC accumulated over 500% less of ^45^Ca^2+^ (533%  ± 163%, *n* = 3, *p* < 003, *t*-test). Therefore, one can conclude that since 4 mA induced the highest calcium accumulation, there was no damage to synaptosomes by 3 mA current. The effect of DC stimulation was not limited to the duration of the stimulation but extended at least 6 hrs beyond the period of exposure. As depicted in Figure 4(b), DC-stimulated synaptosomes still accumulated 400% more calcium which was added 6 hrs after finishing the stimulation (*p* < 0.003, *t*-test, compared to nonstimulated controls tested after 6 hrs with experimental samples). Apparently, the accumulation of calcium occurred at least partially via calcium channels since it was significantly attenuated by 5 mM cobalt (*p* < 0.001, *t*-test, Figure 4(b)). Extracellular positively charged calcium ions are attracted by negatively charged molecules of sialic acid which is a major component of glycocalyx. Enzymatic partial removal of sialic acid with neuroaminidase significantly enhanced DC-induced calcium accumulation (Figure 5(a), *p* < 0.006, *t*-test).

### 3.5. The Influence of Na^+^ and Size of the Electrodes on DC-Induced Calcium Trafficking

**Figure 6:**
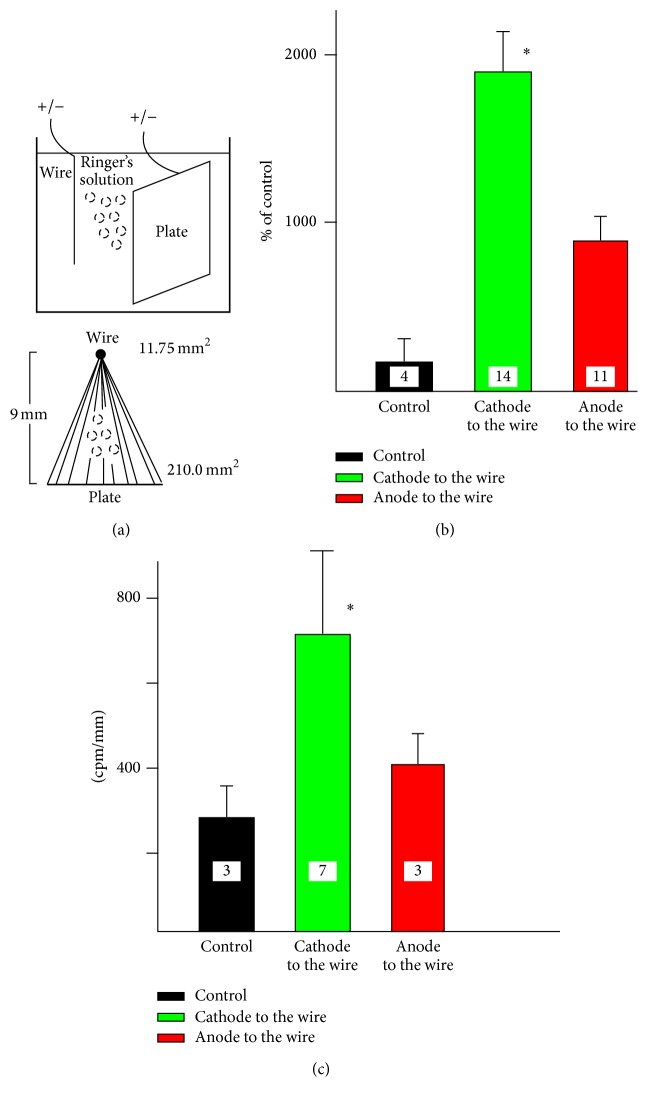
Accumulation of ^45^Ca^2+^ by synaptosomes and segments of the sciatic nerve stimulated with two electrodes of different areas. (a) An experimental setup. The stainless steel plate (210 mm^2^) was inserted into the plastic tube at the distance of 9 mm from the stainless steel wire (11.75 mm^2^). The preparations (synaptosomes or segments of the sciatic nerve attached to the wooden stick) were placed in the tube and stimulated with DC (3′, 3 mA). The anode or cathode of DC was connected to either the wire or the plate. The broken lines in the lower panel of the figure illustrate hypothetical flow of the current. (b) The accumulation of ^45^Ca^2+^ by synaptosomes, while anode (black) or cathode (red) was connected to the wire. (c) The accumulation of ^45^Ca^2+^ by the segments of the sciatic nerve, while anode (black) or cathode (red) was connected to the wire; ^*∗*^ *p* < 0.001; Mann–Whitney* U* test.

The change in the ionic environment of the preparation by omitting Na^+^ ions also affected ^45^Ca^2+^ accumulation. The osmolarity of the incubation solution remained the same since sodium ions were replaced with equivalent concentration of choline chloride. The synaptosomal suspension accumulated more ^45^Ca^2+^ in the absence of sodium. The effect was relatively minor although statistically significant even without any electrical stimulation. However, application of DC to synaptosomes in sodium-free environment almost tripled the amount of accumulated ^45^Ca^2+^ (Figure 5(b)). In the experiments described so far, each preparation was stimulated by two electrodes of identical size. In order to differentiate between the effects of anodal and cathodal stimulation, synaptosomes and segments of sciatic nerve were stimulated in vitro by exposure to DC generated by two electrodes of different sizes (Figure 6(a)). The connection of anode or cathode to the smaller electrode (wire) generated more intense anodal or cathodal current, respectively. The lower panel in Figure 6(a) illustrates the relative size of both electrodes and hypothetical lines and density of flowing current. Although both polarities enhanced accumulation of ^45^Ca^2+^ by synaptosomes, cathodal stimulation was much more effective (Figure 6(b), ^*∗*^ *p* < 0.001, Mann–Whitney* U* test). The stimulation of the segment of sciatic nerve with two electrodes of different sizes yielded results very similar to stimulation of synaptosomal suspension (Figure 6(c)). The application of the cathodal current to the wire resulted in much greater concentration of radioactivity in the nerve (^*∗*^ *p* < 0.001, Mann–Whitney* U* test) than anodal current. It has to be emphasized that, in the case of both of these preparations, namely, synaptosomes and sciatic nerve, the results represent sole accumulation of ^45^Ca^2+^ as it was impossible to measure any translocation of accumulated calcium in this experimental arrangement.

## 4. Discussion

Considering the goal of our experiments, the employment of radioactive calcium seemed to be more appropriate than widely used calcium imaging [41]. Firstly, we wanted to compare three preparations: two in vitro and one in vivo. Considering technical challenges of calcium imaging [41], it has been concluded that using relatively simple but quantitative method for radioactive calcium determination will yield the data which can be compared with higher confidence. Secondly, calcium indicators have their own calcium-buffering abilities influencing free calcium concentration [42]. Undoubtedly, the employment of ^45^Ca^2+^ seems to be much less invasive and damaging for cytoplasm than calcium imaging. Additionally, using radioactive calcium overcame the problem arising from limited dynamics of the radiometric imaging (saturation of different probes depending on their *K*_*d*_ values).

Finally, even a slight change in free calcium concentration due to calcium-induced calcium release may hypothetically obscure the data collected with calcium imaging procedures [50] more than that with radioactive tracer. Thus, it has been decided that in spite of its drawbacks the method of using ^45^Ca^2+^ would be the most suitable one to achieve objectives of intended experiments.

Our method provided information about DC-induced, bulk relocation of calcium within an axon or between external environment (an incubation medium) and internal compartment of the stimulated neuronal preparation. Although one can be tempted to assume that an increase in radioactivity inside neuronal preparation is due to elevation in calcium accumulation, it may not be the only explanation. The DC-induced increase in internal radioactivity can only be the result of radioactive calcium accumulation. This can reflect an increase in radioactive and exogenous, nonradioactive calcium accumulation, an increase in the radioactive and endogenous calcium exchange, or combination of both of these processes occurring simultaneously during and/or after stimulation. Regardless of the interpretation, it is apparent that calcium metabolism and homeostasis were significantly modified by DC stimulation. The evaluation of ^45^Ca^2+^ accumulation and relocation does not allow us to determine the destination of the calcium entering the cell and the cellular origin of the endogenous calcium which may exchange with radioactive ions. Nevertheless, we are convinced that the most of the observed increase in radioactivity results from increase in radioactive calcium accumulation. The rise in the exchange rate of endogenous/radioactive calcium would likely be significantly hampered by relatively slow diffusion of calcium [51, 52] within cytoplasm of the axon. In contrast, the DC-induced movement of ^45^Ca^2+^ calcium observed in our preparations was massive and relatively fast. Additionally, we also observed an increase in radioactivity when ^45^Ca^2+^ was added 6 hrs after the stimulation. If the increase in radioactive calcium would be the result of exchange, it would take place during stimulation and would most likely end before subsequent addition of ^45^Ca. Therefore, since an increase was still observed 6 hrs later, it was probably due to persistent change in channels, carriers, or pumps responsible for the transport of calcium across the cellular membranes. The DC-induced increase in relocation of ^45^Ca^2+^ in our preparations was dramatically reduced by cobalt, a general blocker of calcium channels. We believe that this reinforces our original assumption that the intracellular increase in radioactive calcium is a result of increased accumulation, not just an exchange, although our data do not allow us to draw any conclusions about possibility and intensity of this process. Nevertheless, this underlines the advantage of our technique which permits, in opposite to radiometric measurements, the determination of calcium source in this type of experiments. In conclusion, one can strongly advocate the idea that involvement of exchange process is likely to be minimal since it would involve relatively fast relocation of calcium from different intracellular compartments and move it out of the cell.

Our data in support of in vivo experiments on brain tissue [44], and human keratinocytes [53], convincingly show that the exposure to DC stimulation enhances accumulation/exchange of calcium by biological preparations. It was observed in vivo with the intact sciatic nerve and in vitro with the segments of sciatic nerve, spinal cord, and synaptosomes. The DC-induced amplification in ^45^Ca^2+^ accumulation was much lower in the segments of the sciatic nerve and spinal cord than in synaptosomes. This difference is most likely related to considerably higher accessibility of the membranes of synaptosomes as compared to axons of the spinal cord surrounded by meninges and sciatic nerve embedded in epineurium. It also indicates that DC-induced increase in synaptosomal fraction is mostly due to calcium accumulation, not binding to external surface of synaptosomes, especially that it was attenuated by the presence of cobalt, which blocks most of the calcium channels [54], and was almost abolished by rupture of synaptosomes by osmotic shock [47]. Much greater accumulation of ^45^Ca^2+^ by synaptosomes than by other preparations may be also related to geometry of these structures. Synaptosomes represent symmetrical, spherical vesicles which accumulate calcium into a single limited space without the ability to redistribute it along its length, while sciatic nerve and spinal cord are elongated preparations allowing entering calcium to be relocated along their long axis further away from the point of entry. This was clearly evident in sciatic nerve in vitro, where radioactive calcium was detected in the inside segments, as well as the outside segment and even in the solution of the chamber containing outside segment. This indicated that the forces responsible for redistribution of calcium were strong enough to draw it even outside of the tissue. Although the spinal cord was too fragile for this type of experiments, we assume that redistribution of calcium would be similar to what was observed in the sciatic nerve.

The increase in the strength of stimulation from 1 to 4 mA was followed by a gradual statistically significant enhancement in ^45^Ca^2+^ accumulation (Figure 4(a)). The trend of enhanced ^45^Ca^2+^ accumulation parallel to the increased strength of the stimulation was clear, although the difference between 1 and 2 mA was not statistically significant. The increase in calcium accumulation was observed when the calcium was present during stimulation and was also recorded when calcium was added several hours after termination of the stimulation. Although both anodal and cathodal stimulations were similarly effective with both electrodes of the same size, cathodal stimulation produced greater change when one of the electrodes was much larger than the other. While the concentration of radioactive calcium in our preparations was in the range of 3-4 nM, the concentration of endogenous calcium in the axon is in the range of 40–100 nM [55, 56]. Due to nearly 35-fold dilution of radioactive calcium, the accumulation/relocation of 1 nM of ^45^Ca^2+^ observed in our experiments would reflect relocation of approximately 35 nanomoles of endogenous calcium. One has to also realize that the effect of DC-induced relocation of calcium to the cytoplasm, where most of the calcium targets are located, can be significantly influenced by endoplasmic reticulum and mitochondria [56]. While the concentration of endogenous cytoplasmic calcium is in the range of 100 nM, micromolar and even millimolar concentrations are observed in endoplasmic reticulum and mitochondria, respectively. Those two intracellular compartments have the ability to buffer intracellular calcium concentration reducing its toxicity [27, 56] and could sway DC-induced elevation in calcium trafficking. Our data supports and extends the results obtained in vivo by Islam and collaborators [44] who demonstrated that anodal stimulation applied in either single (30 *μ*A, 30 min) or repetitive (5 times every 24 hrs) paradigms of stimulation induced an increase in ^45^Ca^2+^ accumulation. The increase in vivo [44] was not the same in all brain structures and yielded damaged cells after repeated stimulations. Considering very high accumulation of ^45^Ca^2+^ induced by 4 mA current (Figure 4(a)) and very low accumulation by osmotically damaged synaptosomes, it seems very unlikely that there was any DC-induced damage of synaptosomes, even by 4 mA. Since in Islam et al.'s [44] in vivo experiments and in some of our experimental design the increase in calcium accumulation and relocation was also observed when it was applied after cessation of DC stimulation, it is clear that not only DC itself but also the processes initiated by its application (e.g., trafficking of channels and receptors, relocation of internal organelles [57, 58]) persist beyond the period of stimulation and contribute to the observed effects. Our data obtained with sciatic nerve also unequivocally indicate that, as previously reported [59], not only the trafficking but also electrophoretically induced [60] redistribution of the calcium following its entrance to the preparation was modified by DC stimulation. This has been demonstrated by the experiments employing segments of sciatic nerve in vivo and in vitro. In both cases, the increase in calcium accumulation was noticed not only in the segments directly bathed in ^45^Ca^2+^ but also in the adjacent areas. Calcium entering the axon is being relocated by DC to the adjacent segment of the sciatic nerve and even out of the axon to the extracellular Ringer's solution. This is particularly discernible in vitro in the case of anodal stimulation. One can speculate that positively charged, anodal electrode pushed calcium ions into the axon, while those ions were simultaneously pulled by negative, cathodal electrode on the other side of the partition resulting in the elevation of calcium concentration in the outer segment and even in extracellular Ringer's solution in the outer chamber. The inner segment under the anode would have ^45^Ca^2+^ ions moving towards the cathode, away from inner segment. This repulsive action of anode towards positive calcium ions would be amplified by negativity of the entire outer segment. The negativity of the outer segment could even attract some radioactive calcium ions to Ringer's solution in the outer chamber (indeed, there is a slight increase). The cathode under inner segment would make the inner segment negative. This negativity combined with repulsive action of anode under the outer segment would push ^45^Ca^2+^ ions into the nerve, but the movement of ions towards the outer segment would be attenuated by positivity of the anode under outer segment. Thus, the increase in ^45^Ca^2+^ accumulation in the outer segment may be reduced by positivity of the entire outer segment. Therefore, in the case of cathodal stimulation, there was enhanced calcium accumulation in the inner segment but insignificant calcium redistribution. Hence, application of DC clearly enhanced calcium accumulation and polarity-dependent redistribution within the axon. The tangible content of ^45^Ca^2+^ in the axon in vivo could be influenced by bidirectional exchange of axonal ^45^Ca^2+^ with the interstitial fluid. Some calcium could diffuse back from the nerve to the interstitial fluid. It was technically impossible to measure this amount since it would be immediately diluted by the mixture of the interstitial fluid and Ringer's solution added to keep the preparation moist. As demonstrated by our previous electrophysiological experiments [8], this rapid calcium relocation could affect the amplitude of the compound action potential (CAP) recorded in vivo from DC-stimulated nerve [8]. The redistribution of calcium observed in current experiments and alterations in the amplitude of CAP [8] were both clearly related to the polarity and the strength of applied DC current. Moreover, the time frame of DC exposure required for both types of the changes to become apparent (1–3 min) was almost identical. Those correlations constitute a crucial link between DC-induced modulation of CAP and cellular background of this modulation observed as a change in the free calcium concentration/relocation inside of stimulated tissues.

Since movement of radioactive calcium is very slow following its injection into squid axon [61, 62], it is most likely driven by diffusion without participation of the axonal transport. In our experiments, anodal and cathodal stimulations significantly accelerated calcium relocation, most likely through electroosmosis in the axons and Schwann cells [63]. Since changes in DC-induced calcium redistribution were immediately apparent, it is unlikely that they resulted from the modification of the axonal transport. However, delayed, long-lasting effects of DC exposure on the axonal transport cannot be excluded and are worthy of further research. Although our results were obtained on sciatic nerve, the rate of movement would be most likely similar in other components of the nervous system.

Considering changes in calcium ionic activity following injury to the nervous system [64, 65], consequences of an increase in intracellular calcium concentration during and following DC exposure can be massive. As a second messenger, calcium ions are involved in several processes crucial for neuronal physiology including recovery of neuronal tissue from traumatic injury [5, 9, 25]. The involvement of calcium in neuronal excitability, synaptic activity and plasticity, extension of filopodia, formation of new synaptic contacts, guidance of synaptic sprouting [31], and receptor trafficking represents only a partial list of processes regulated by an increase in intracellular calcium concentration. The exposure to DC stimulation and subsequent free calcium elevation can alter not only biochemistry of the cell but also its morphology [59, 60]. The exposure of neurons in culture to DC changes the location of intracellular organelles which favor cathode during their repositioning [60]. Those changes which also included polarization of the entire cell were evident within 1 hr of exposure and determined the migratory pattern of neurons during subsequent development [59]. As DC exposure dramatically enhances calcium trafficking through the membranes, it becomes an extremely potent tool which can significantly alter neuronal action [4, 6, 8, 9, 25].

The incubation of synaptosomes with neuroaminidase which removes sialic acid from extracellular glycoproteins and gangliosides modified DC-induced ^45^Ca^2+^ accumulation. Sialic acid, which contributes to charged cloud on the cellular surface generated by glycocalyx, is especially important for nervous system function. It participates in neuroglial interactions [66], synaptic plasticity [67], and excitability [68] and is a structural component of sodium [69, 70] and calcium [71] channels. Remarkably, removal of sialic acid significantly alters exchange of ions in several biological preparations [72]. As evident from our data, depleting sialic acid and subsequent change in the charge of the membrane noticeably amplify ability of the calcium to penetrate through this modified membrane. As cobalt, known blocker of presynaptic calcium channels [46], reduced calcium accumulation, we propose that at least partial enhancement of the DC-induced calcium penetration through the membrane occurs via calcium channels. Alternative explanation could be offered assuming that charged calcium channels [72] could be persistently modified/activated after being electrophoretically relocated in the membrane by DC as reported for other functional proteins [70, 72]. Interestingly, an elevation in ^45^Ca^2+^ accumulation was also observed in the experiments conducted in Na^+^-free Ringer's solution. There was over 200% (233.3%) more ^45^Ca^2+^ in synaptosomes stimulated in Na^+^-free Ringer's solution than in control suspension stimulated in the presence of sodium. Moreover, in agreement with previously reported data [73], nonstimulated controls accumulated 25.5% more ^45^Ca^2+^ in Na^+^-free environment. The calcium concentration in neurons is regulated by plasma membrane Ca^2+^ATPase and Na^+^/Ca^2+^ exchanger [73–75]. While Ca^2+^ATPase is more involved in calcium homeostasis, Na^+^/Ca^2+^ exchanger counteracts significant changes in calcium concentration to prevent its toxicity [75]. The concentration of calcium inside of preparations evaluated in our experiments is apparently the results of dynamic equilibrium established by all processes which are forcing calcium in and out of intracellular space. It is clear that DC stimulation is shifting this equilibrium towards calcium accumulation/exchange which then exceeds exclusion of intracellular calcium. In support of this notion, we observed, as mentioned above, increased ^45^Ca^2+^ accumulation in Na^+^-free environment. Clearly, reduced Na^+^ concentration affects the DC-modulated dynamics of calcium equilibrium. One can speculate that Na/Ca exchanger [74, 75] removes accumulated ^45^Ca^2+^ less effectively when the counterion (Na^+^) is missing. Alternatively, it is tempting to suggest that DC stimulation opens calcium channels and there is a massive influx of  ^45^Ca^2+^ into the synaptosomes. This massive influx and subsequent enhancement in intracellular calcium concentration stimulate compensatory activity of Na/Ca exchanger which increases pumping of calcium out in an attempt to readjust calcium concentration to its original equilibrium. However, in the absence of Na^+^ the activity of this exchanger is attenuated and calcium entering synaptosomes during DC stimulation remains inside. Apparently, the Na/Ca exchanger works also without DC stimulation, since control synaptosomes in Na^+^-free Ringer's solution accumulated more ^45^Ca^2+^ than controls in Ringer's solution-containing sodium ions. Remarkably, treatment with neuroaminidase has similar effect. This would suggest that the presence of sialic acid is essential for full activity of the Na/Ca exchanger.

The geometry and size of the electrodes critically influenced the data. One can assume that the density of the current is inversely related to the surface area of the electrodes. Therefore, using electrodes of different sizes which would subsequently generate polarized currents of uneven strength would help to determine which of the two polarities is more effective. In stimulated sciatic nerve and synaptosomes (Figure 6), cathodal current was much more efficient than anodal in increasing calcium accumulation. While stimulation of synaptosomes with two electrodes of the same size (Figure 4(a), 3 mA) amplified accumulated radioactivity approximately 1,200-fold, cathode connected to the wire increased it by almost 2,000-fold (Figure 6(c)). This observation may be vitally important for future clinical applications of DC stimulation. The issue related to the geometry of electrodes is the relation between the electrodes and spatial position of the neurons in stimulated preparation. This factor may play very different role in synaptosomes, sciatic nerve, and the spinal cord. As mentioned before, the synaptosomes represent spherical structure. One can assume that the applied current would influence all of them in a very similar way. On the contrary, the nerve was stimulated by perpendicular current and the application of DC parallel to the axis of the axon could have different still unknown consequences. While long motor neurons run in the spinal cord parallel to its long axis, short interneurons may be spatially arranged in a variable way. Therefore, our experiments with the spinal cord represent the model reminiscent of the experimental arrangement used in vivo experiments to stimulate brain [44]. In both cases, stimulated tissue demonstrated increased concentration of ^45^Ca^2+^ which persisted beyond the period of stimulation.

While application of DC is an emerging and very formidable procedure to persistently modify function of the nervous system [76], one has to realize that DC-induced redistribution of calcium ions is an indicative of electrophoretic processes which occur within the axon under the influence of direct current. Other ions, charged molecules, subcellular structures, and even some organelles can be redistributed under the influence of this current as well [62]. As such, if used in clinical setting, DC stimulation has to be applied with high attentiveness. It has been demonstrated that excessive calcium accumulation may be detrimental for cell physiology [56]. Also, the enhanced calcium accumulation can exert quite opposite effects on neuronal plasticity depending on the rate of calcium accumulation and its final concentration induced by physiological process or experimental procedure [37].

## Figures and Tables

**Table 1:** Neuronal preparations and statistical tests used to evaluate DC-induced calcium relocation.

Type of experiment	Statistical test	Probability
*(i) Axon in vivo*		
Anodal versus cathodal	One-way ANOVA followed by Dunn's test	*p* < 0.001
Anode in versus control in	Mann–Whitney *U* Rank Sum test	*p* < 0.008
Cathode in versus control in	Mann–Whitney *U* Rank Sum test	*p* < 0.03
Cathode versus anode (combined segments)	Mann–Whitney *U* Sum Rank test	*p* < 0.001
*(ii) Axon in vitro*		
Inner and outer chambers versus “anode in”	Mann–Whitney* U* Sum Rank test	*p* < 0.036
Inner and outer segments combined versus control	Mann–Whitney *U* Sum Rank test	*p* < 0.001
*(iii) Spinal cord in vitro*	*t*-test	*p* < 0.029
*(iv) Synaptosomes*		
^45^Ca present during stim	ANOVA followed by Holm-Sidak	*p* < 0.001
^45^Ca added 6 hrs after stim	*t*-test	*p* < 0.003
^45^Ca in the presence of cobalt	*t*-test	*p* < 0.001
^45^Ca/neuroaminidase/stim versus no neuroaminidase/stim	*t*-test	*p* < 0.006
Na^+^ versus no Na^+^, no stim	ANOVA followed by Dunn's test	*p* < 0.001
Na^+^ versus no Na^+^, stim.	*t*-test	*p* < 0.029
Osmotic shock	*t*-test	*p* < 0.003
Different electrodes	Mann–Whitney *U* Sum Rank test	*p* < 0.001
